# Adverse Hemodynamics in Kommerell's Diverticulum: A Comparative Case Report Linking Oscillatory Shear Index to Aortic Dissection

**DOI:** 10.1002/ccr3.72509

**Published:** 2026-04-12

**Authors:** Kenji Sakakibara, Hiroyuki Nakajima, Yukiyo Yoshida, Yudai Hagihara, Chie Nakamura, Soshi Yamamoto, Daichi Shikata, Yuki Takesue, Satoru Shiraiwa, Yoshihiro Honda, Shigeaki Kaga, Masahiro Hamasaki, Hisashi Johno, Yu Sasaki, Hiroaki Watanabe

**Affiliations:** ^1^ Department of Surgery (II), Faculty of Medicine University of Yamanashi Chuo Japan; ^2^ Department of Radiology, Faculty of Medicine University of Yamanashi Chuo Japan

**Keywords:** aortic diseases, aortic dissection, blood flow velocity, cardiovascular models, cine magnetic resonance imaging, computer simulation, hemodynamics, thoracic

## Abstract

Hemodynamic evaluation using four‐dimensional flow magnetic resonance imaging or computational fluid dynamics can identify a high‐risk phenotype in Kommerell's diverticulum, which is characterized by intradiverticular vortex formation, low wall shear stress, and elevated oscillatory shear index (OSI). This functional assessment provides crucial risk stratification beyond anatomical size, as a high OSI may pinpoint intimal vulnerability sites and potential dissection entry.

## Key Clinical Message

Hemodynamic analysis using 4D Flow magnetic resonance imaging or computational fluid dynamics can identify a high‐risk Kommerell's diverticulum phenotype characterized by vortex flow, low wall shear stress, and high oscillatory shear index, enabling risk stratification beyond anatomical size, as a high oscillatory shear index may indicate sites of intimal vulnerability.

**Abbreviations**
4D Flow MRI: four‐dimensional flow magnetic resonance imaging
AAo: ascending aorta
ASCA: aberrant subclavian artery
CFD: computational fluid dynamics
CT: computed tomography
DAo: descending aorta
KD: Kommerell's diverticulum
LCCA: left common carotid artery
LSA: left subclavian artery
OSI: oscillatory shear index
RCCA: right common carotid artery
RSA: right subclavian artery
TAVI: transcatheter aortic valve implantation
TEVAR: thoracic endovascular aortic repair
WSS: wall shear stress

## 1 Introduction

Kommerell's diverticulum (KD), an outpouching at the origin of an aberrant subclavian artery arising from a right‐sided or variant aortic arch, is a rare but clinically significant anomaly that can lead to dysphagia, aneurysm enlargement, aortic dissection, and rupture. Conventional risk assessment primarily relies on morphological indices, such as maximal diameter; nonetheless, outcomes vary widely, and size alone does not fully reflect the biological vulnerability of the lesion.

Emerging evidence implicates abnormal hemodynamics, particularly spatial and temporal fluctuations in wall shear stress (WSS) and the oscillatory shear index (OSI), in aortic disease pathogenesis. A high OSI, representing bidirectional shear fluctuation during the cardiac cycle, has been associated with sites prone to intimal injury. Four‐dimensional (4D) flow magnetic resonance imaging (MRI) enables noninvasive, in vivo, time‐resolved, three‐directional velocity mapping, whereas computational fluid dynamics (CFD) provides high‐resolution, patient‐specific simulations.

Here, we present a comparative two‐case analysis combining CFD and 4D flow MRI to determine whether a consistent hemodynamic “risk phenotype” exists in KD.

## 2 Case Presentation

### 2.1 Case History/Examination

#### 2.1.1 Case 1

A man in his 60s presented to the emergency department with sudden severe tearing chest and back pain radiating to the interscapular region. He had a history of long‐standing hypertension with suboptimal control, but no known connective tissue disorder. On arrival, he was hypertensive and diaphoretic. Peripheral pulses were symmetric, and there were no focal neurological deficits.

#### 2.1.2 Case 2

**FIGURE 1:**
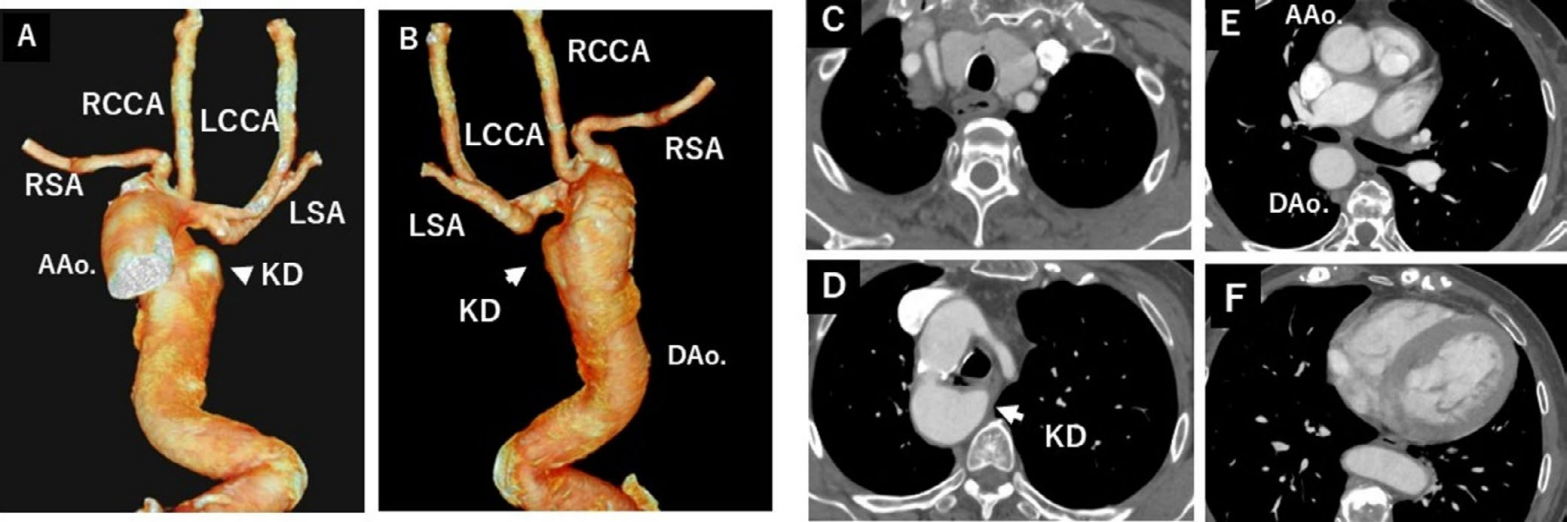
Computed tomography (CT) in Case 2. (A, B) Three‐dimensional volume‐rendered CT images from anterior and posterior views, respectively, demonstrating a right aortic arch with a large Kommerell's diverticulum (KD, indicated by arrowhead/arrow). (C–F) Axial CT images demonstrating the anatomy of the aortic arch and the KD (arrowhead in D).

An 80‐year‐old woman presented with dyspnea. Cardiac echocardiography revealed severe aortic valve stenosis, and transcatheter aortic valve implantation (TAVI) was planned. Preprocedural chest imaging performed for noncardiac evaluation incidentally revealed a right‐sided aortic arch with a large KD (Figure 1). The patient denied dysphagia, chest pain, and hoarseness. Her blood pressure was well controlled with oral medication, and there were no clinical features suggestive of connective tissue disease.

### 2.2 Differential Diagnosis, Investigations, and Treatment

#### 2.2.1 Case 1

**FIGURE 2:**
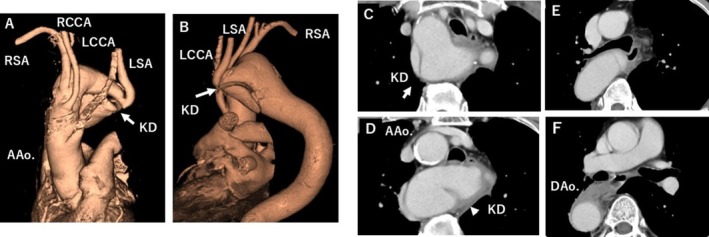
Computed tomography (CT) in Case 1. Demonstrating aortic dissection. (A, B) Three‐dimensional volume‐rendered CT images from anterior and posterior views, respectively, showing a right aortic arch with a Kommerell's diverticulum (KD, arrows). (C–F) Axial CT images showing the KD and the intimal flap of the aortic dissection (arrowhead in D and E). Abbreviations: AAo., ascending aorta; DAo., descending aorta; RCCA, right common carotid artery; LCCA, left common carotid artery; RSA, right subclavian artery; LSA, left subclavian artery.

The initial differential diagnoses included acute aortic syndrome, acute coronary syndrome, pulmonary embolism, and esophageal rupture. Electrocardiography showed sinus rhythm without ST‐segment elevation, and high‐sensitivity troponin levels were normal. Contrast‐enhanced computed tomography (CT) angiography demonstrated a right aortic arch with KD at the origin of an aberrant left subclavian artery. Importantly, CT revealed acute aortic dissection with a primary entry tear arising directly from the KD (Figure 2). There was no clinical or radiographic evidence of visceral or limb malperfusion.

An urgent aortic repair was performed. Given the location of the tear and the arch anatomy, the patient underwent total arch replacement with insertion of a frozen elephant trunk to exclude the KD. In addition, embolization of the left subclavian artery and revascularization of the involved subclavian circulation were performed. Completion angiography confirmed complete exclusion of the KD and entry tear, with brisk antegrade flow through the arch.

A retrospective CFD analysis was performed to investigate the hemodynamic characteristics underlying the KD phenotype. The CFD analysis was outsourced to Cardio Flow Design Inc. (Tokyo, Japan) [1]. Patient‐specific geometries were reconstructed from thin‐slice, early‐phase contrast‐enhanced multidetector‐row CT data (DICOM format). Segmentation and three‐dimensional model generation were performed using OsiriX (OsiriX Foundation, Geneva, Switzerland), followed by surface editing and smoothing in 3D‐Coat (PIGWAY, Kyiv, Ukraine). Computational meshes were generated in ANSYS‐ICEM CFD 16.2 (ANSYS Japan, Tokyo, Japan) with > 2,000,000 cells using tetrahedral elements and three boundary‐fitted prism layers to improve near‐wall resolution for WSS and OSI estimation.

### 2.3 Boundary Conditions

To allow the development of inlet velocity profiles and to approximate flow development around the aortic valve, the ascending aortic inlet was extruded to five times its diameter. A pulsatile mass‐flow inlet waveform was prescribed with cardiac output set at 5.0 L/min. Outlet boundaries for the supra‐aortic branches, descending aorta, and bilateral coronary arteries were extended to 50 times each vessel diameter to stabilize flow splitting on the basis of prior validation of the aortic CFD workflow. Pressure outlet boundary conditions were applied to reflect extra‐domain forces including wave reflection. Coronary outlets were modeled as pulsatile mass‐flow boundaries, assigning 2.5% of total aortic flow to each coronary artery (total coronary flow = 5% of cardiac output). Vessel walls were assumed rigid.

### 2.4 Flow Solver and Postprocessing

Unsteady flow simulations were performed in ANSYS‐FLUENT 16.2 (ANSYS Japan, Tokyo, Japan) by solving the incompressible transient Navier–Stokes equations with Newtonian blood properties (density 1060 kg/m^3^; dynamic viscosity 0.004 kg/(ms)). Given a peak systolic Reynolds number of approximately 4000, turbulence was modeled using the RNG k–ε scheme. The time step was set to 1 × 10^−5^ s to maintain a sufficiently low Courant number, and convergence criteria were set to residuals < 1 × 10^−5^ at each time step. Hemodynamic metrics including flow vectors, WSS, and OSI were computed. OSI was defined as:

$$\mathrm{OSI}=0.5\left(1-\frac{|\int_{0}^{T}{\vec{\tau }}_{w}\left(t\right)\;\mathrm{dt}|}{\int_{0}^{T}|{\vec{\tau }}_{w}\left(t\right)|\mathrm{dt}}\right),$$

where ${\vec{\tau }}_{w}$ denotes the WSS vector and T is the cardiac cycle duration.

**FIGURE 3:**
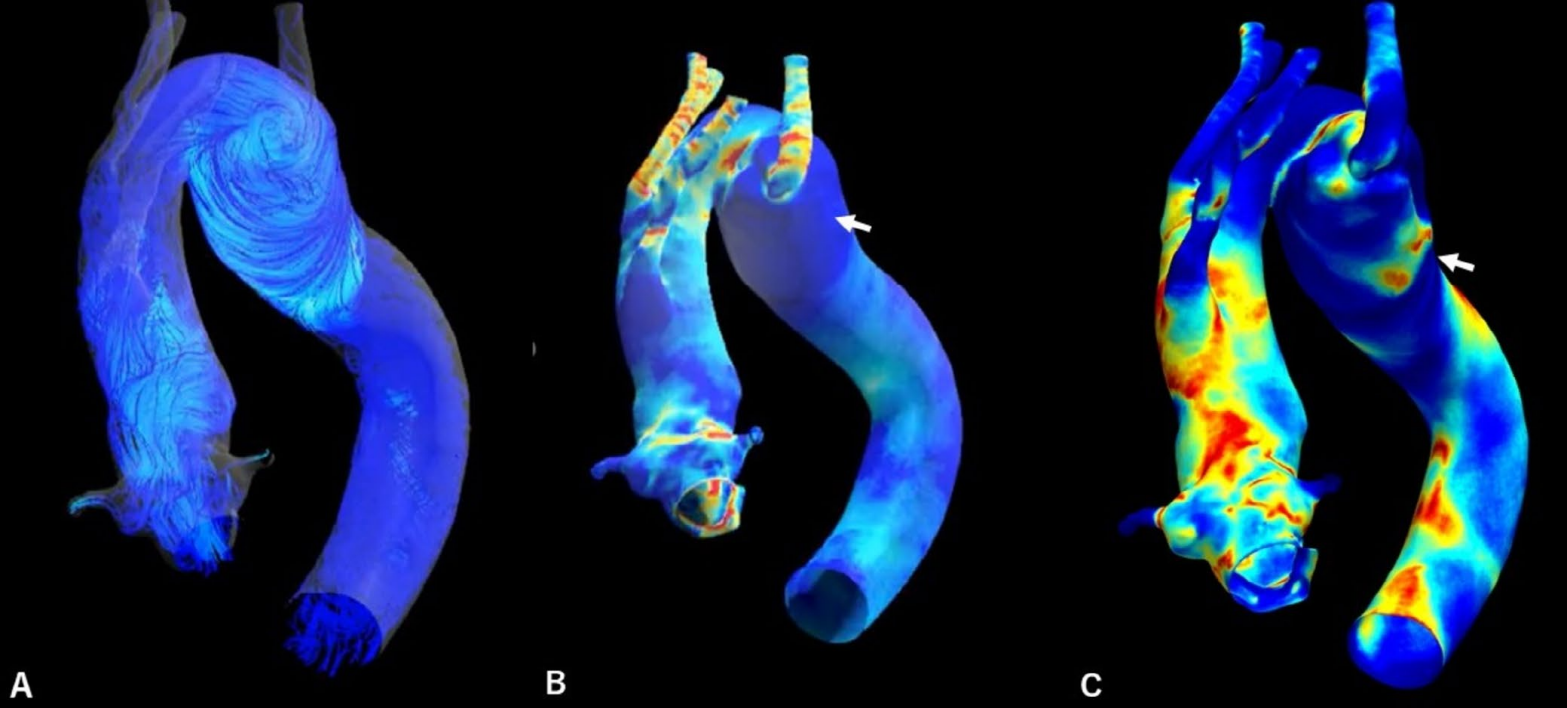
Computational fluid dynamics (CFD) analysis in Case 1. CFD analysis was performed on a pre‐dissection model reconstructed from CT data. (A) Blood flow velocity streamlines reveal a large vortex within the Kommerell's diverticulum (KD). (B) Wall shear stress (WSS) map exhibits diffusely low WSS (blue areas). (C) Oscillatory shear index (OSI) map. The white arrow indicates a region of high OSI on the posterior‐to‐inferior wall of the KD, which corresponded closely to the actual site of the dissection entry tear.

Postprocessing demonstrated three key findings: (i) a large, stable intradiverticular vortex, (ii) diffusely low time‐averaged WSS across the KD wall, and (iii) a focal area of high OSI (approaching the upper range of 0.5) concentrated at the KD ostium and a small region within the sac. Notably, the peak OSI region on the posterior‐to‐inferior KD wall was spatially aligned with the clinically inferred entry tear (Figure 3, Video 1).

**VIDEO 1:** Computational fluid dynamics (CFD) analysis in Case 1. This video demonstrates the simulated hemodynamics within the right aortic arch and the Kommerell's diverticulum (KD) over a representative cardiac cycle based on a pre‐dissection model. The animated velocity streamlines clearly illustrate the formation and persistence of a large swirling vortex within the KD throughout the cycle. This abnormal flow pattern results in fluctuating shear forces on the aortic wall, leading to the creation of a focal area with high oscillatory shear index (OSI). This high‐OSI region corresponds precisely to the location where the primary entry tear of the aortic dissection subsequently occurs, highlighting the predictive value of hemodynamic analysis. Video content can be viewed at https://onlinelibrary.wiley.com/doi/10.1002/ccr3.72509.

The authors provided the source imaging data and reviewed the modeling assumptions and outputs used in this report.

#### 2.4.1 Case 2

The main clinical question was whether this asymptomatic KD was suitable for conservative surveillance or whether it posed a higher risk of future aneurysmal enlargement or dissection/rupture.

**FIGURE 4:**
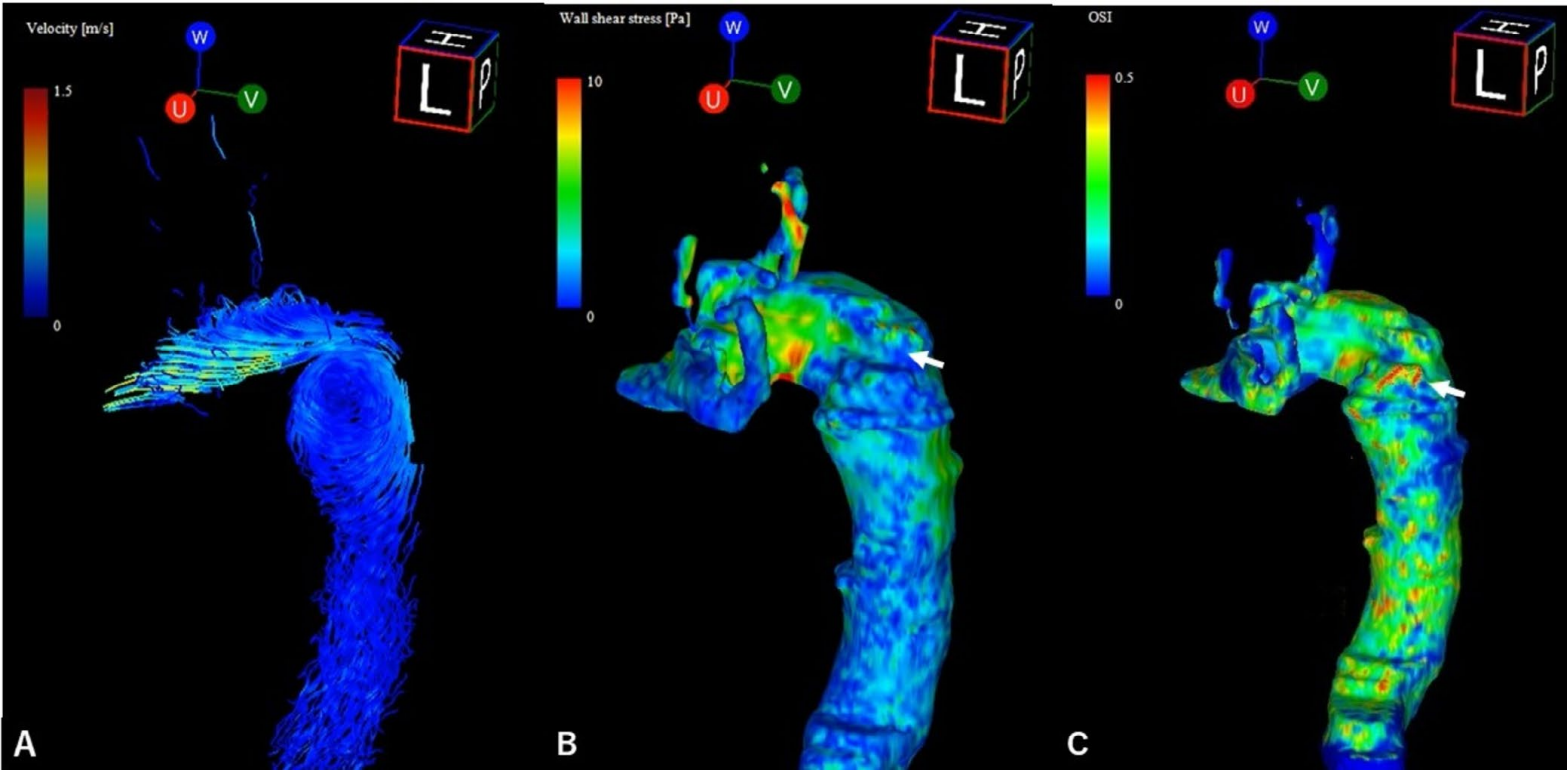
Four‐dimensional (4D) flow magnetic resonance imaging (MRI) analysis in Case 2. (A) Velocity streamlines visualize complex, vortical blood flow within the Kommerell's diverticulum (KD). (B) Wall shear stress (WSS) map shows diffusely low WSS. (C) Oscillatory shear index (OSI) map. The white arrow highlights a localized area of elevated OSI on the wall of the KD, indicating a potential hemodynamic risk zone.

To refine risk assessment beyond anatomical size, whole‐chest 4D flow MRI was performed using a 3‐Tesla scanner with time‐resolved, three‐directional velocity encoding. After standard preprocessing, hemodynamic analysis was conducted using dedicated software (iT‐Flow2; Cardio Flow Design, Tokyo, Japan). The in vivo findings closely mirrored the computational hemodynamics observed in Case 1. Specifically, the analysis revealed a prominent intradiverticular vortex on path line visualization, diffusely reduced WSS along the KD wall, and focal OSI elevation centered at the diverticular inlet (Figure 4, Video 2). This pattern of adverse hemodynamics in an asymptomatic patient without overt morphological complications suggested a preexisting “at‐risk” hemodynamic phenotype.

**VIDEO 2:** Four‐dimensional (4D) flow magnetic resonance imaging (MRI) analysis in Case 2. This video shows in vivo blood flow dynamics measured within the right aortic arch and Kommerell's diverticulum (KD) in an asymptomatic patient. Time‐resolved velocity streamlines reveal a prominent helical and vortical flow pattern that develops within the KD as blood is ejected from the heart during systole. This disturbed flow persists through diastole. The analysis identifies a region of elevated oscillatory shear index (OSI) on the diverticulum wall, indicating a zone of high hemodynamic stress. These findings demonstrate the utility of 4D flow MRI in identifying potential high‐risk areas for future aortic events, such as aneurysm formation or dissection. Video content can be viewed at https://onlinelibrary.wiley.com/doi/10.1002/ccr3.72509.

The patient underwent TAVI for severe aortic valve stenosis. However, a postoperative decline in activities of daily living and worsening dementia were noted. Therefore, close follow‐up with serial CT was selected rather than prophylactic aortic intervention.

### 2.5 Outcome and Follow‐Up

#### 2.5.1 Case 1

The postoperative course was uncomplicated, and the patient was discharged on guideline‐directed antihypertensive therapy. At 24‐month follow‐up, CT angiography confirmed persistent exclusion of the KD and entry tear, with false‐lumen thrombosis adjacent to the repair and no evidence of endoleak or need for reintervention. The patient remained asymptomatic and normotensive.

#### 2.5.2 Case 2

During 12 months of post‐TAVI surveillance, no aortic enlargement or dissection was observed, and the patient remained under close observation.

Overall, despite the different clinical settings and imaging modalities, both cases demonstrated a consistent adverse hemodynamic pattern characterized by intradiverticular vortex formation, low WSS, and focal high OSI, suggesting the presence of a shared KD risk phenotype.

## 3 Discussion

This comparative two‐case analysis identified a consistent and potentially high‐risk hemodynamic pattern in KD, characterized by the triad of intradiverticular vortex formation, low WSS, and OSI focal elevation. The striking spatial co‐localization of the peak OSI with the entry tear in the dissected case (Case 1) provides compelling evidence that a high OSI may represent a key biomechanical factor promoting intimal vulnerability in KD. Pathophysiologically, OSI induces a pro‐inflammatory and pro‐thrombotic endothelial phenotype, potentially predisposing the aortic wall to injury and degeneration. This finding aligns with the results of previous CFD studies that have successfully implemented hemodynamic parameters to predict future aortic dissection sites in other contexts [1]. Furthermore, recently, Cheng et al. demonstrated that helical vortex flow originating from an aberrant right subclavian artery can directly induce dissection tears, providing a strong mechanistic link to our vortex formation observation [2].

Replication of the same hemodynamic triad by in vivo 4D flow MRI in an asymptomatic patient (Case 2) is a crucial finding. Therefore, adverse flow conditions may represent an inherent KD anatomy feature and can exist long before the clinical symptom or morphological change development, such as aneurysmal dilatation, thereby highlighting a significant limitation of current surveillance strategies, which are based primarily on diameter and may fail to identify hemodynamically “at‐risk” lesions [3]. The association between variant arch anatomy and aortic dissection is well established [4], and our findings offer a potential hemodynamic mechanism to explain this clinical association. By identifying a pre‐dissection “risk phenotype,” we may better stratify patients who appear stable according to anatomical criteria alone.

These findings are particularly relevant to the existing literature. A recent systematic review by Loschi et al., encompassing 426 patients with an aberrant subclavian artery, reported a 3.8% rupture rate and KD diameters ranging widely from 12.6 mm to 63.6 mm, underscoring the clinical heterogeneity and diameter‐based threshold limitations for intervention [5]. The repair mortality rates were similar across different modalities, emphasizing the importance of appropriate patient selection for intervention [5]. Hemodynamic indices, such as OSI mapping, may supplement anatomic measurements for a more individualized risk stratification, aiding correct timing and intervention type.

From a methodological perspective, both CFD and 4D flow MRI have emerged as powerful tools for analyzing aortic blood flow [6, 7]. While CFD relies on certain assumptions and 4D flow MRI resolution depends on the acquisition parameters, the convergence of their findings in our two cases strengthens the validity of the observed hemodynamic pattern. Notably, our group has previously utilized 4D flow MRI to successfully analyze complex flow dynamics in other aortic pathologies, including dissection with an aberrant right subclavian artery and distal stent graft‐induced new entry, further supporting the robustness of this noninvasive technique [8, 9]. Our findings support a pragmatic framework in which 4D flow MRI or CFD is used to identify a KD “risk phenotype” (vortex + low WSS + high OSI). High OSI foci, particularly near the diverticular ostium, could justify more intensive surveillance or earlier intervention, moving beyond a “one‐size‐fits‐all” approach.

In this study, we utilized two distinct but complementary methods: CFD for high‐resolution retrospective analysis and 4D flow MRI for noninvasive in vivo evaluation. While we did not perform a direct head‐to‐head comparison of the two modalities in the same individual, the emergence of an identical hemodynamic triad (vortex, low WSS, and high OSI) across different patients and different modalities strongly supports the validity of this risk phenotype. Previous studies have reported a good correlation between 4D flow MRI and CFD in aortic pathologies [10], and our findings further suggest that both tools are clinically valuable for identifying hemodynamically vulnerable KD lesions.

### 3.1 Limitations

There are some limitations to this study. First, a direct comparison between CFD and 4D flow MRI in the same patient was not performed. Although the findings were qualitatively identical across the two cases, future studies involving simultaneous validation with both modalities in a larger cohort are warranted to quantify the degree of agreement between computational simulations and in vivo measurements.

## 4 Conclusion

Despite different clinical states and imaging modalities, KD may exhibit a characteristic adverse hemodynamic pattern consisting of vortex formation, low WSS, and focal high OSI. Co‐localization of high OSI with dissection entry tears in our index case strongly suggests that OSI is a promising risk stratification biomechanical marker. Noninvasive 4D flow MRI enables visualization of these otherwise silent hemodynamic stresses. Incorporating functional imaging into clinical practice may substantially enhance decision‐making for surveillance and intervention, moving beyond purely morphology‐based risk assessment for patients with KD.

## Author Contributions

**Kenji Sakakibara:** conceptualization, writing – original draft. **Hiroyuki Nakajima:** supervision, writing – review and editing. **Yukiyo Yoshida:** investigation, writing – review and editing. **Yudai Hagihara:** investigation. **Chie Nakamura:** investigation. **Soshi Yamamoto:** writing – original draft. **Daichi Shikata:** investigation. **Yuki Takesue:** investigation. **Satoru Shiraiwa:** investigation. **Yoshihiro Honda:** investigation. **Shigeaki Kaga:** software, writing – review and editing. **Masahiro Hamasaki:** formal analysis, methodology, supervision. **Hisashi Johno:** investigation, software, supervision. **Yu Sasaki:** investigation, software. **Hiroaki Watanabe:** formal analysis, investigation, software, supervision.

## Funding

This work was supported by JSPS KAKENHI Grant Number (25K12088 25K19134).

## Consent

The patient provided written informed consent for the publication of this case report.

## Conflicts of Interest

The authors declare no conflicts of interest.

## Data Availability

All data regarding this case has been reported in the manuscript. Please contact the corresponding author if you are interested in any further information.
